# Enhancement of sperm quality and fertility-related parameters in Hubbard grandparent rooster fed diets supplemented with soybean lecithin and vitamin E

**DOI:** 10.1016/j.psj.2021.101635

**Published:** 2021-12-09

**Authors:** Shahram Shabani, Morteza Mehri, Fatemeh Shirmohammad, Mohsen Sharafi

**Affiliations:** ⁎ Department of Animal Science, Shahr-e-Qods Branch, Islamic Azad University, Tehran, Iran; † Department of Animal Science, Faculty of Agriculture, Tarbiat Modares University, Tehran, Iran

**Keywords:** vitamin E, soybean lecithin, sperm motility

## Abstract

The purpose of this study was to investigate the effect of dietary supplementation of different levels of soybean lecithin and vitamin E on semen quality parameters and some reproductive hormones in Hubbard grandparent roosters. The experiment was conducted in a 3 × 2 factorial arrangement with 3 levels of soybean lecithin (0, 1, and 2%) and 2 levels of vitamin E (0 and 300 mg/kg). Semen samples were collected on d 0, 20, 40 and 60 of the experiment and analyzed. Adding 1% soybean lecithin and vitamin E into the diet increased semen volume and sperm concentration, membrane integrity and viability (*P* < 0.05). Supplementing diets with 1 or 2% lecithin in addition to vitamin E significantly improved total motility and progressive motility (*P* < 0.05). Vitamin E significantly increased the amplitude of lateral head displacement (**ALH**) of sperm (*P* < 0.05). Although there was no effect on LH and FSH when diets were supplemented with vitamin E and 1 or 2% lecithin, testosterone concentration was increased (*P* < 0.05). Malondialdehyde (**MDA**) concentration was significantly lower in all 3 treatments containing vitamin E (*P* < 0.05). It can be concluded that supplementation of rooster diets with vitamin E and 1% lecithin can improve fertility related parameters in Hubbard grandparent roosters.

## INTRODUCTION

Semen characteristics and sperm function are major parameters determining rooster fertility potential. These features might be affected by many physiological and environmental factors such as age, diet, stress, and disease (Sikka, 1996). Age is one of these critical factors because it comes with several physiological changes that result in oxidative stress and the production of a huge amount of reactive oxygen species (**ROS**) which has negative impacts on sperm structure and function. A typical example of aging in poultry industry is a considerable reduction in semen quality and reproductive potential of broiler breeder roosters after 45 wk of age, which is a typical response to aging (Raei et al., 2021). As above mentioned, there are many reasons for this decline, most notably is considering ROS as a factor increasing the accumulation of ROS in the body which is concomitant with reduced semen antioxidant capacity (Weil et al., 1999). Moreover, as a consequence of oxidative stress, membrane lipid peroxidation leads to initiate the programmed death known as apoptosis, and consequently decrease the fertility potential (Partyka et al., 2010; Bansal and Bilaspuri, 2011).

One of the strategies that can reduce detrimental effects of oxidative stress is dietary manipulation or incorporation of lipid sources or antioxidants into diet in order to reduce oxidative stress (Safari Asl et al., 2018). Lipid sources such as fatty acids and phospholipids can increase the membrane fluidity and flexibility of sperm and increase its resistance against oxidative stress. Also, they contribute to several crucial enzymatic pathways involved in antioxidant regulations and steroidogenesis (Nakamura et al., 2004). Antioxidants can neutralize ROS either inside or outside of the cell; reducing apoptosis and inhibit ROS formation (Bansal and Bilaspuri, 2011).

Soybean lecithin is used as a source of phospholipids in either diets or semen extenders (Papa et al., 2010). Although dietary effects of soybean lecithin have been assessed in other species, it has not been considered for grandparent roosters. In vitro trials have shown that adding lecithin into semen extenders improved membrane fluidity, increased motility, and preserved acrosome integrity of sperm in a variety of domestic animals (Akhter et al., 2011; Crespilho et al., 2012; Emamverdi et al., 2013). Lecithin increases the plasma membrane fluidity of sperm as well as signal transduction pathways that consequently improve the fertility potential (Sun et al., 2021). Lecithin can also provide the antioxidant properties; Lecithin inclusion in the diet of rats reduced intestinal inflammation and oxidative stress by decreasing lipid peroxidation, as well as improving antioxidant defenses (Colares et al., 2016). Dietary usage of 6% soybean lecithin in the layer hen diet was shown to improve hatchability and fertility (Attia et al., 2009). Inclusion of soybean lecithin at 0.5, 1, and 1.5% in the rabbit diet also improved sperm quality characteristics (Attia and Kamel, 2012).

Given the fact that lecithin is considered as a lipid source, it is always necessary to protect its structure against peroxidation. When natural or synthetic antioxidants are used as a supplement to protect lipids in the diet or semen extenders, it was accompanied by an improvement in sperm quality (Behnamifar et al., 2020). As of yet, a variety of antioxidants have been used with lipid sources or with lecithin (Surai et al., 2002). Vitamin E is one of the strongest antioxidants and has been shown to have protective effects on sperm. This potential was first identified in the semen of turkeys and later in the semen of other birds such as roosters (Rengaraj and Hong, 2015). By alleviating oxidative stress (Min et al., 2016) and inhibiting lipid peroxidation in sperm, vitamin E effectively prevents intracellular free radicals and protects the viability and integrity of sperm membranes (Fujihara and Howarth, 1978). Therefore, the purpose of this study was to consider the effects of adding soybean lecithin and vitamin E into the diet on sperm quality characteristics and some reproductive hormones in Hubbard grandparent roosters.

## MATERIAL AND METHODS

### Chemicals

All chemicals used in this study were obtained from Sigma (St. Louis, MO) and Merck (Darmstadt, Germany). Soybean lecithin was purchased from Jahan Shimi Company (Tehran, Iran), and vitamin E was purchased from Bahavand Daru Company (Tehran, Iran).

### Birds, Housing, and Experimental Diets

Experimental procedures were approved by the Islamic Azad University Ethics Committee. In order to evaluate the effects of soybean lecithin and vitamin E on reproductive parameters of Hubbard grandparent males, thirty-six, 45-wk-old, Hubbard M99, Line A roosters, with a mean weight of 4,480 g and a CV of 10.1%, were randomly distributed into 6 treatments, with 6 replicates and each individual rooster was the experimental unit. The experiment was conducted in a 3 × 2 factorial arrangement in a completely randomized design with 3 levels of soybean lecithin (0, 1, and 2%) and 2 levels of vitamin E (0 and 300 mg/kg). The roosters were housed in individual cages (70 cm × 70 cm × 85 cm) and kept in 18 to 22°C and 15L:9D light schedule. Ad libitum water was provided for roosters during the experiment. The control group was fed a basal diet (Table 1). The isoenergetic and isonitrogenous diets were formulated to meet the nutritional recommendations of the Hubbard M99 grandparent management guide (Table 1).

**Table 1:** Ingredients and the composition of diets fed to Hubbard M99 Line A grandparent roosters.

	Diets*		
Ingredients (g/kg)	Basal diet	Lecithin (1%)	Lecithin (2%)
Corn	600	600	600
Soybean meal (44% CP)	72.8	71.5	70.4
Wheat	72.0	40.7	30
Barley	90	90	62
Wheat bran	126.5	148.9	178.8
Premix⁎⁎	6	6	6
Oyster shell	14.8	15.2	15.4
Dicalcium phosphate	13.3	12.5	11.8
DL-methionine	0	0.5	0.5
Common salt	4.6	4.6	4.6
Lecithin	0	10	20
Contents by calculation			
ME (kcal/kg)	2,800	2,800	2,800
CP (%)	12.22	12.20	12.22
Calcium (%)	0.90	0.90	0.90
Available P (%)	0.39	0.39	0.39
Methionine (SID) (%)	0.26	0.26	0.26
Lysine (SID) (%)	0.49	0.49	0.49
TSAA (SID) (%)	0.46	0.46	0.46

⁎ To prepare the diets containing vitamin E, 300 mg of vitamin E/kg was added to these diets.

⁎⁎ Supplied the following per kg of diet: vitamin A, 13,200 IU; vitamin D3, 4,200 IU; vitamin E, 120 IU; vitamin K3, 6 mg; niacin, 66 mg; riboflavin, 14.4 mg; thiamin, 3.0 mg; pantothenic acid, 18 mg; folic acid, 2.4 mg; pyridoxine, 4.8 mg; vitamin B12, 0.04 mg, and biotin, 0.3 mg. Fe (FeSO_4_•H_2_O), 60 mg; Mn (MnSO_4_•H_2_O), 148 mg; Zn (ZnO), 120 mg; Cu (CuSO_4_•5H_2_O), 12 mg; iodine (KI), 2.4 mg; and Se (Na_2_SeO_3_), 0.36 mg.

### Semen Collection and Semen Quality Assessment

Semen collection was performed by the abdominal massage method described by Burrows and Quinn (1937) on d 1, 20, 40, and 60 to cover at least 3 consecutive spermatogenesis cycles. Evaluation was performed individually on the mentioned days.

### Volume

Semen volume was measured using graduated collecting tubes. An aliquot of distilled water and semen dilution of 1:200 was placed on a Neubauer hemocytometer for determination of sperm concentration (Shahverdi et al., 2015).

### Motion Characteristics

Motility characteristics of sperm were measured using Sperm Class Analysis software (SCA; Version 5.1; Microptic, Barcelona, Spain). Semen samples were diluted with PBS (1:10) and 5 µL of semen were delivered onto a prewarmed chamber slide (38°C, Leja 4; 20 mm height; Leja Products, Luzernestraat B.V., Holland) (Shahverdi et al., 2015). Sperm kinematic values included curvilinear velocity (**VCL**), straight line velocity (**VSL**), average path velocity (**VAP**), the amplitude of lateral head displacement (**ALH**), and linearity (LIN=VSL/VCL × 100) and straightness (STR= VSL/VAP × 100) were also recorded.

### Viability

To measure sperm viability, the Annexin V-FITC kit (IQP, Groningen, the Netherlands) was used for the determination of externalization of phosphatidyl serine as an indicator of apoptotic-like changes in the semen. Sperm samples were washed in calcium buffer, and the concentration was readjusted to 1 × 10^6^ sperm/mL, and 10 µL of Annexin-V was added to 100 µL of the sample. After the incubation of the samples for 15 min at room temperature, 1 μL of Propidium Iodide (**PI**) was added to each sample, and the sample was evaluated using a flow cytometer (Lotfi et al., 2017).

### Plasma Membrane Integrity

The sperm plasma membrane integrity was determined using the Hypoosmotic Swelling (**HOS**) Test (Lotfi et al., 2017). In this test the sperm is exposed to a hypoosmotic solution. The osmolality of the HOS medium is 100 mOsm/L, and the osmolality required for sperm is 425 mOsm/L. As a result, the tails of normal sperm will swell when exposed to this solution (a low-osmolarity environment), whereas damaged sperm with low motility will not swell measurably. For the current experiment, 5 µL of semen was mixed with 50 µL of hypoosmatic solution (100 mOsm/L, 57.6 mM fructose, and 19.2 mM sodium citrate). After 30 min of incubation, the sperm samples were checked under a phase-contrast microscope (E200, Nikon, Tokyo, Japan).

### Morphology

To evaluate sperm morphology and calculate the percentage of abnormal sperm, a Hancock solution was used (Najafi et al., 2019). To evaluate total sperm abnormalities, at least 10 μL of each semen sample were pipetted into 1 mL of Hancock's solution (62.5 mL of 37% formalin, 150 mL of sodium saline solution, 150 mL of PBS buffer, and 500 mL of distilled water). Then 10 µL of this solution was put on a warm slide and the percentage of total sperm abnormalities (head abnormalities, detached heads, abnormal mid-pieces, and tail defects) was recorded by counting a total of 400 sperm under a phase-contrast microscope (E200, Nikon, Tokyo, Japan) with magnification of 1,000.

### DNA Fragmentation

The sperm chromatin structure assay (**SCSA**) procedure was used to determine DNA damage in spermatozoa using flow cytometry analysis (Evenson and Wixon, 2005). An aliquot of washed spermatozoa in PBS was diluted to a concentration of 3 ×$10^{6}$ spermatozoa/mL. The cell suspension was treated with an acid detergent solution contained 0.1% Triton X-100, 0.15 mol/L NaCl, and 0.08 N HCl for 30 s, and then stained with 6 mg/L purified acridine orange (**AO**) in a phosphate-citrate buffer. The AO binds to single-stranded DNA and emits a red fluorescence that is detected using a 670 bandpass filter (Fl-3). The percentage of DNA fragmentation in spermatozoa was calculated by DNA fragmentation index obtained from the ratio of red cells to the total of red green cells (Hosseinifar et al., 2015).

### Lipid Peroxidation

The concentration of Malondialdehyde (MDA) as an indication of lipid peroxidation in the semen was tested. A spectrophotometer (1200-UV, Shimadzu, Japan) was used to numerate the amount of MDA molecule reacting with 2 molecules of thiobarbituric acid (**TBA**), resulting in the maximum absorption at 532 nm (Esterbauer et al., 1991).

### LH, FSH, and Testosterone Concentrations

Blood samples were drawn from the brachial vein at d 1 and 60 (10–11 AM) to determine the concentrations of LH, FSH, and testosterone hormones. The blood was then centrifuged for 10 min at 4°C at 3,000 rpm to separate the plasma. The plasma concentrations of the hormones were measured using a chicken ELISA kit and as prescribed by the manufacturer (Nanjing, Jiangsu, China) (Teymouri Zadeh et al., 2020).

### Statistical Analysis

Prior to data analysis, normality and homogeneity of variances of the data were assessed using the Shapiro-Wilk and Levene's test with the UNIVARIATE procedure, respectively. Arc-sine transformation was also performed on percentage data, if needed. The analysis was done by PROC MIXED for repeated measurements and PROC GLM for single observations using SAS 9.1 (SAS Institute Inc., Cary, NC). Further analysis of statistical differences between the treatments was conducted using Tukey-Kramer test. In all statistical analyses, the significance level was set at *P* < 0.05.

## RESULTS AND DISCUSSION

This study investigates the effects of supplementation of diet with lecithin and vitamin E on sperm characteristics and reproductive hormones in Hubbard grandparent roosters. Sperm characteristics which were measured in this study were total motility, progressive motility, straight-line velocity (VSL), curvilinear velocity (VCL), average pass velocity (VAP), straightness (STR), linearity (LIN), and amplitude of lateral head displacement (ALH) were shown in Table 2.

**Table 2:** Effect of lecithin and vitamin E on sperm characteristics of Hubbard M99 Line A grandparent roosters.

		Total motility (%)	Progressive motility (%)	VSL (μm/s)	VCL (μm/s)	VAP (μm/s)	STR (%)	LIN (%)	ALH (μm)
Treatments^1^									
Lecithin, %	Vitamin E, ppm								
0	0	86.04^b^	40.82^ab^	73.35	181.61	42.65	54.89	40.16	7.70
0	300	86.79^b^	37.83^b^	74.80	184.48	42.19	52.60	40.32	8.23
1	0	86.20^b^	38.44^b^	74.22	184.81	44.14	53.88	39.84	7.83
1	300	90.46^a^	43.91^a^	74.44	181.37	40.68	54.49	40.32	8.17
2	0	85.31^b^	39.07^b^	73.26	181.03	42.82	54.58	40.00	7.95
2	300	90.10^a^	43.40^a^	73.67	184.54	44.22	55.29	39.52	8.32
SEM Pooled		1.476	1.758	2.478	2.898	1.775	1.447	1.607	0.509
Main effects									
Lecithin, %									
0		86.42^b^	39.33	74.08	183.05	42.42	53.75	40.32	7.97
1		88.33^a^	41.18	74.33	183.08	42.41	54.19	40.16	8.00
2		87.71^ab^	41.24	73.47	182.77	43.52	54.94	39.84	8.14
Vitamin E, ppm									
0		85.85^b^	39.44^b^	73.61	182.48	43.20	54.45	40.00	7.83^b^
300		89.12^a^	41.71^a^	74.30	183.45	42.36	54.13	40.00	8.24^a^
*P*-value									
Lecithin		0.047	0.067	0.698	0.984	0.359	0.266	0.811	0.746
Time		0.209	0.234	0.140	<0.0001	0.001	0.0004	0.0001	<0.0001
Vitamin E		<0.0001	0.004	0.426	0.529	0.255	0.593	0.949	0.041
Lecithin × Time		0.035	0.071	0.190	0.368	0.185	0.048	0.392	0.364
Lecithin × Vitamin E		0.022	<0.0001	0.829	0.141	0.073	0.083	0.805	0.906
Vitamin E × Time		0.011	0.001	0.058	0.466	0.094	0.586	0.051	0.241
Lecithin × Vitamin E × Time		0.440	0.054	0.251	0.027	0.112	0.345	0.294	0.258

Data are presented as means ± SEM (n = 6 roosters/each treatment).

Superscripts with different means within column differ significantly at *P* ≤ 0.05.

1 Roosters received their diets at 45 wk of age for 60 d based on experimental design as follows: 1) basal diet, 2) basal diet supplemented with 300 mg/kg vitamin E, 3) basal diet supplemented with 1% lecithin, 4) basal diet supplemented with 1% lecithin along with 300 mg/kg vitamin E, 5) basal diet supplemented with 2% lecithin, and 6) basal diet supplemented with 2% lecithin along with 300 mg/kg vitamin E. Abbreviations: ALH, amplitude of lateral head displacement; LIN, linearity; STR, straightness; VAP, average path velocity; VCL, curvilinear velocity; VSL, straight-line velocity.

**Figure 1:**
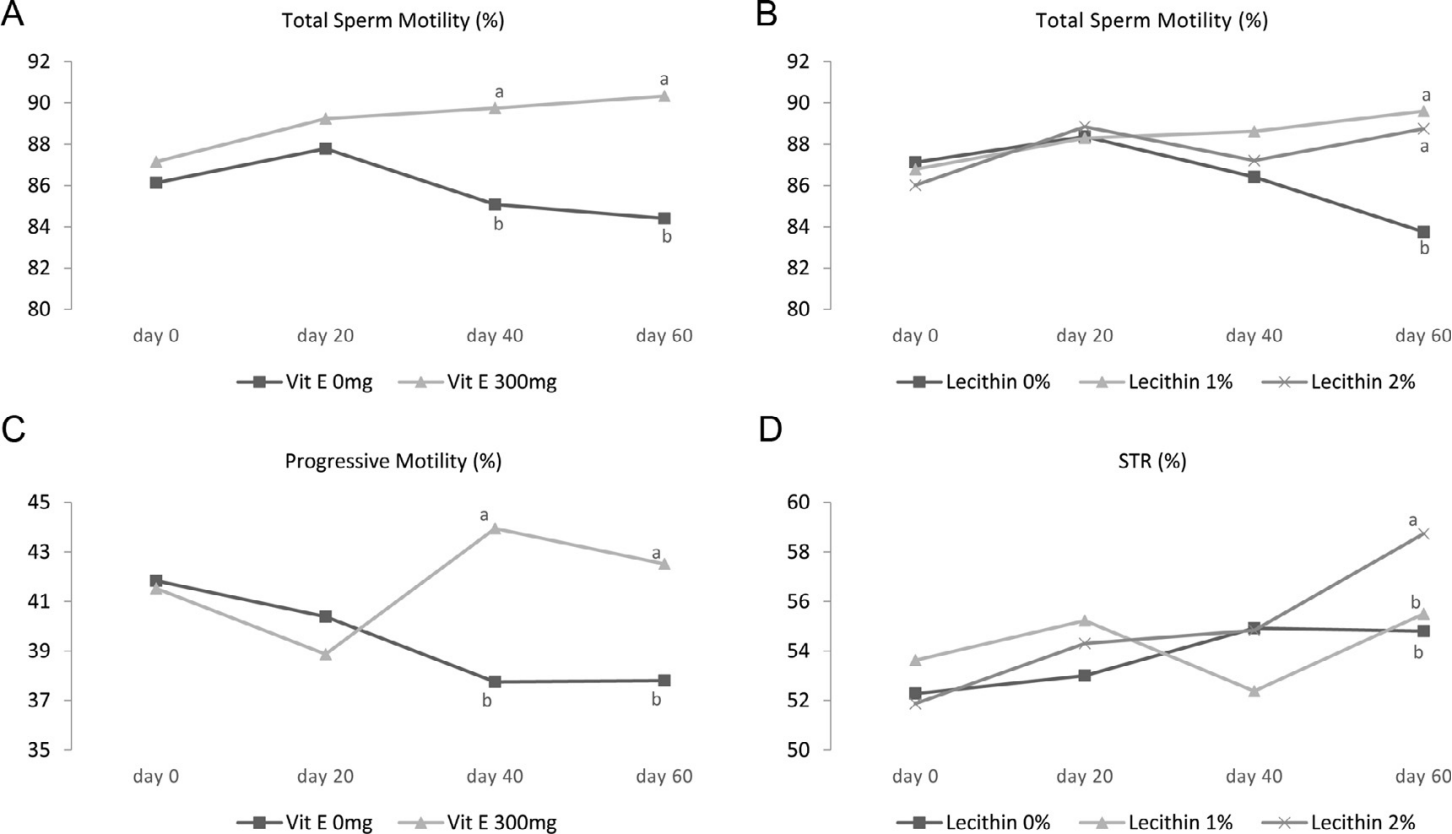
Effect of vitamin E × time interaction on total sperm motility (A) and on progressive motility (B); and lecithin × time interaction on total sperm motility (C) and on STR (D) in 45-wk-old Hubbard M99 Line A grandparent roosters fed diets for 60 d. Total sperm motility, progressive motility and STR were measured by sperm class analysis software (SCA). Superscripts on different means within each day differ significantly at *P* ≤ 0.05.

The results showed that 1 or 2% lecithin along with vitamin E improved significantly total motility compared to the control group or the groups that received only vitamin E or lecithin treatments (*P* < 0.05). The same trend was observed for progressive motility with the exception that the groups treated with vitamin E + lecithin did not show a significant difference with the control group (*P* < 0.05). Figures 1A to C show the significant interaction effect of vitamin E × time and lecithin × time on total motility and progressive motility, respectively (*P* < 0.05). Adding vitamin E into the diet increased total motility at 40 and 60 d of the experiment and adding lecithin into the diet improved this trait at 60d of the experiment (*P* < 0.05). Figure 1C also demonstrates the effect of vitamin E on a significant increase in progressive motility at 40 and 60d of the experiment (*P* < 0.05). The inclusion of vitamin E and lecithin to the diet as well as their interaction resulted in no significant effect on VCL, VSL, VAP, STR, and LIN (*P* > 0.05). As shown in Figure 1D, the interaction effect of lecithin × time on STR was significant when 2% lecithin was included into the roosters’ diet at 60d (*P* < 0.05). Adding vitamin E to the roosters' diet increased significantly the ALH (*P* < 0.05) but different levels of lecithin did not affect this trait. This improvement in motion characteristics can have a big impact on the fertility potential of semen (Tsakmakidis, 2010), because there is a significant correlation between motility and fertility (Herrara et al., 2005). This improvement might be attributed to the beneficial effects of either lecithin or vitamin E as they can combat reactive oxygen species (ROS) caused by various external and internal factors (Leão et al., 2021).

To our knowledge this is the first study considering the addition of lecithin and vitamin E into the diets of aged roosters. We found a significant improvement in the semen parameters of rooster under effects of combined lecithin and vitamin E. This improvement agrees with the recent study by Safari Asl et al. (2018) who reported using 0.16 ratio of omega-3 (O3) to omega-6 (O6) fatty acids in the diets of broiler breeder roosters increased semen quality and improved fertility and hatching rates (Safari Asl et al. 2018). Moreover, our findings are supported by Attia and Kamel (2012) who reported supplementation of rabbit diet with 0.5%, 1.0%, and 1.5% soybean lecithin increased total sperm motility compared to the control group. Beneficial effects of lecithin are not only limited in dietary approaches but also it has been approved for the in vitro methods such as adding in the extender and freezing media (Akhter et al., 2011; Crespilho et al., 2012; Emamverdi et al., 2013).

In a recent study, Behnamifar et al. (2020) studied the impact of different levels of alpha lipoic acid supplementation on semen quality and fertility parameters of aged broiler breeder roosters. They showed an increase in semen parameters when alpha lipoic acid was used as supplementation.

In the current study, we observed vitamin E has shown a strong antioxidant capacity because several semen parameters, as well as semen lipid peroxidation, were improved by adding the vitamin E into the diets. Antioxidants like vitamin E aid in sperm motility by decreasing free radicals. Mohamad Asrol and Abdul Rashid (2017) investigated the effect of increasing dietary levels of vitamin E on semen quality and quantity traits of local indigenous roosters and showed improvement in total motility after 4 wk of vitamin E supplementation.

**Figure 2:**
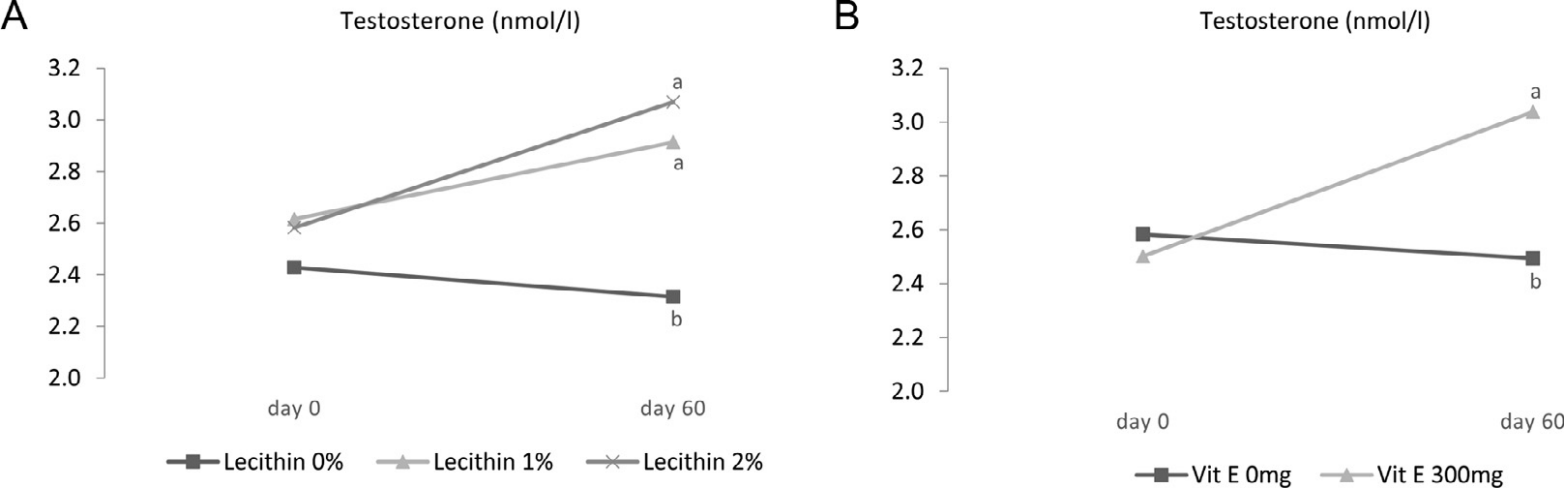
Effect of lecithin × time interaction (A) and vitamin × time interaction (B) on plasma testosterone in 45-wk-old Hubbard M99 Line A grandparent roosters fed diets for 60 d. Testosterone was measured by chicken ELISA kit. Superscripts on different means within each day differ significantly at *P* ≤ 0.05.

Other semen characteristics, including sperm concentration and semen volume as well as the concentration of reproductive hormones such as testosterone, LH, and FSH were also investigated (Table 3). The highest levels of testosterone were observed in roosters received a diet containing lecithin or vitamin E (*P* < 0.05). Both 1 and 2% lecithin levels (Figure 2A) and supplementation with vitamin E (Figure 2B) increased plasma testosterone concentration at 60d (*P* < 0.05). The addition of lecithin and vitamin E to the diet did not lead to changes in LH and FSH concentrations (Table 3). Time and number of samplings in day are considered as important factors that may affect LH levels. This is because of the fact that amplitude and daily fluctuations of gonadotropins are varied at different times of day (Pourazadi et al., 2020). In the current study, there was only a single blood collection from birds each day and its impact might be considerable.

**Table 3:** Effect of lecithin and vitamin E on sperm concentration, semen volume, and plasma sex hormone profile.

		LH (ng/L)	FSH (IU/L)	Testosterone (nmol/L)	Semen volume (mL)	Sperm concentration (1 × 10^9^/mL)
Treatments^1^						
Lecithin, %	Vitamin E, ppm					
0	0	4.04	5.79	2.40^b^	0.51^ab^	2.92^abc^
0	300	3.93	5.71	2.34^b^	0.61^ab^	2.83^bc^
1	0	4.13	6.09	2.52^b^	0.70^a^	2.79^c^
1	300	3.98	5.71	3.01^a^	0.57^ab^	3.25^ab^
2	0	3.87	5.60	2.70^ab^	0.51^ab^	2.88^abc^
2	300	3.94	5.77	2.95^a^	0.41^b^	3.29^a^
SEM Pooled		0.157	0.253	0.145	0.117	0.175
Main effects						
Lecithin, %						
0		3.99	5.75	2.37^b^	0.56^b^	2.88
1		4.06	5.90	2.77^a^	0.64^a^	3.02
2		3.91	5.69	2.83^a^	0.46^c^	3.09
Vitamin E, ppm						
0		4.01	5.83	2.54^b^	0.57	2.87^b^
300		3.95	5.73	2.77^a^	0.53	3.12^a^
*P*-value						
Lecithin		0.296	0.396	<0.0001	0.018	0.096
Time		<0.0001	0.231	0.004	0.135	<0.0001
Vitamin E		0.427	0.460	0.003	0.379	0.003
Lecithin × Time		0.649	0.963	0.006	0.547	0.008
Lecithin × Vitamin E		0.440	0.240	0.013	0.013	0.013
Vitamin E × Time		0.265	0.647	0.0001	0.957	0.0001
Lecithin × Vitamin E × Time		0.491	0.018	0.528	0.069	0.821

Data are presented as means ± SEM (n=6 roosters/each treatment).

Superscripts with different means within column differ significantly at *P* ≤ 0.05.

1 Roosters received their diets at 45 wk of age for 60 d based on experimental design as follows: 1) basal diet, 2) basal diet supplemented with 300 mg/kg vitamin E, 3) basal diet supplemented with 1% lecithin, 4) basal diet supplemented with 1% lecithin along with 300 mg/kg vitamin E, 5) basal diet supplemented with 2% lecithin, and 6) basal diet supplemented with 2% lecithin along with 300 mg/kg vitamin E.

In addition to reduce lipid peroxidation and improving lead-induced histopathological changes by vitamin E, it can increase plasma testosterone levels in the rats (Ayinde et al., 2012). In the line with this statement, in our study, the highest concentration of testosterone was found in the roosters that received a combination of lecithin and vitamin E (*P* < 0.05). Thus, it seems likely that lecithin along with vitamin E, due to having antioxidant properties, decreased oxidative stress, and increased testosterone level.

**Figure 3:**
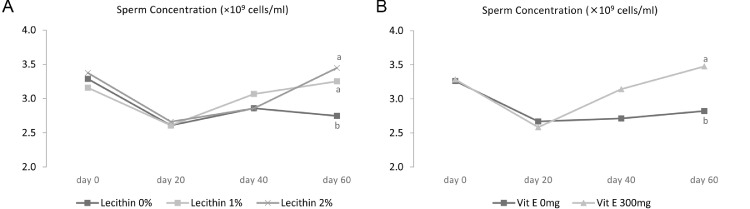
Effect of lecithin × time interaction (A) and vitamin × time interaction (B) on sperm concentration in 45-wk-old Hubbard M99 Line A grandparent roosters fed diets for 60 d. Sperm concentration was measured by Neubauer hemocytometer. Superscripts on different means within each day differ significantly at *P* ≤ 0.05.

The results showed that the combination of vitamin E and 2% lecithin, in comparison to the addition of vitamin E or lecithin alone (at the level of 1%), resulted in a significant improvement in sperm concentration (*P* < 0.05) but, in comparison to the supplementing lecithin alone (1%) resulted in a significant decrease in sperm volume (*P* < 0.05). As demonstrated in Figures 3A and B, regarding sperm concentration, interaction effects of lecithin × time and vitamin × time are significant (*P* < 0.05), which suggest a significant effect of vitamin E and lecithin supplementation for 60 d on sperm concentration of roosters (*P* < 0.05). Cerolini et al. (2006) reported that the addition of 300 mg of vitamin E to the diet of Cobb broiler breeder roosters significantly increased sperm volume and concentration. Similar findings were reported by Darestani et al. (2019) who added 1,000 mg of vitamin E to the Varamini roosters' diet. Our findings in this part of study agree with Attia and Kamel (2012) who investigated the impact of soybean lecithin on rabbit sperm quality, and they found higher semen volume and sperm concentration in the diet supplemented with 1 and 1.5% soybean lecithin. In a same observation in human study by Comhaire et al. (2000), it was found that oral administration of antioxidants such as α-tocopherol significantly increased sperm concentration in oligozoospermic men. Improving semen volume and sperm concentration in the current study seems to be related to improving its oxidative stability of the semen (Wang and Wang, 2008), which appear to be attributed by the addition of soybean lecithin (Attia and Kamel, 2012) or vitamin E (Safari Asl et al., 2018) in the diet of roosters. Nevertheless, some studies have reported contradictory findings. Mohamad Asrol and Abdul Rashid (2017) found that increasing dietary levels of vitamin E had no significant effect on semen volume and sperm concentration. Similar results were reported by Castellini et al. (2003) and Gliozzi et al. (2009) who studied rabbits and found no effect of vitamin E supplementation on sperm volume and concentration.

**Figure 4:**
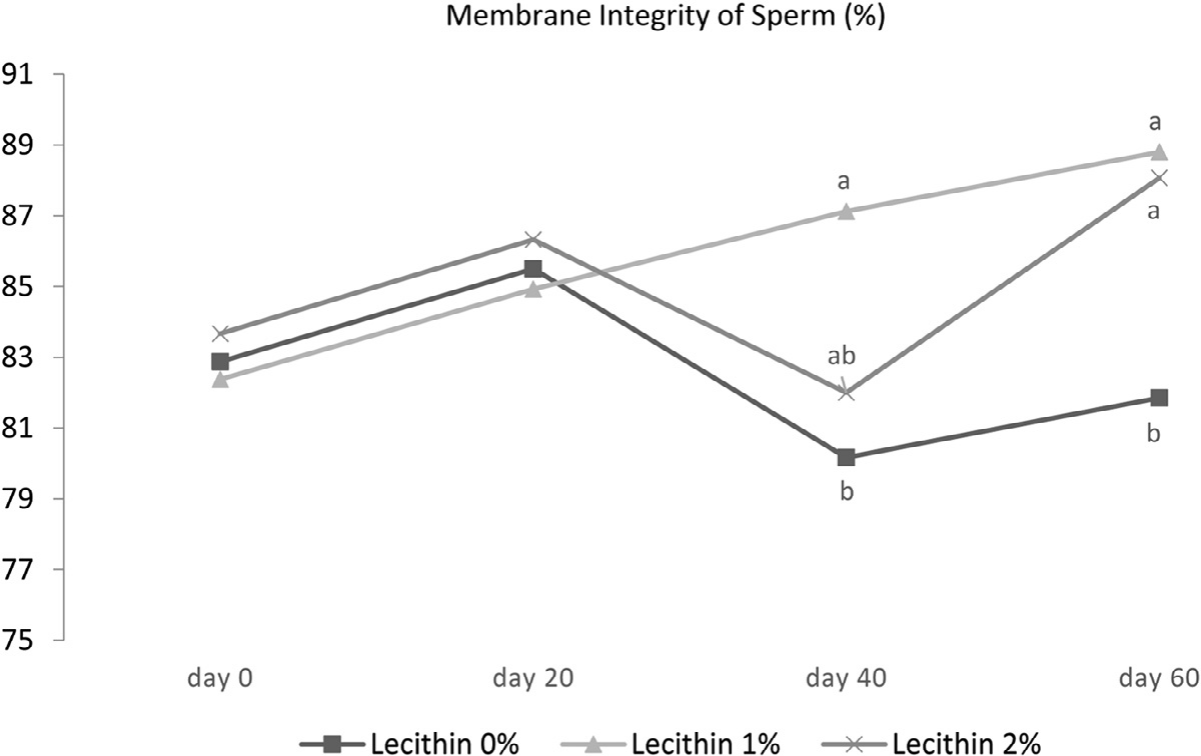
Effect of lecithin × time interaction on membrane integrity in 45-wk-old Hubbard M99 Line A grandparent roosters fed diets for 60d. Membrane integrity was measured by hypo osmotic swelling test (HOST). Superscripts on different means within each day differ significantly at *P* ≤ 0.05.

**Figure 5:**
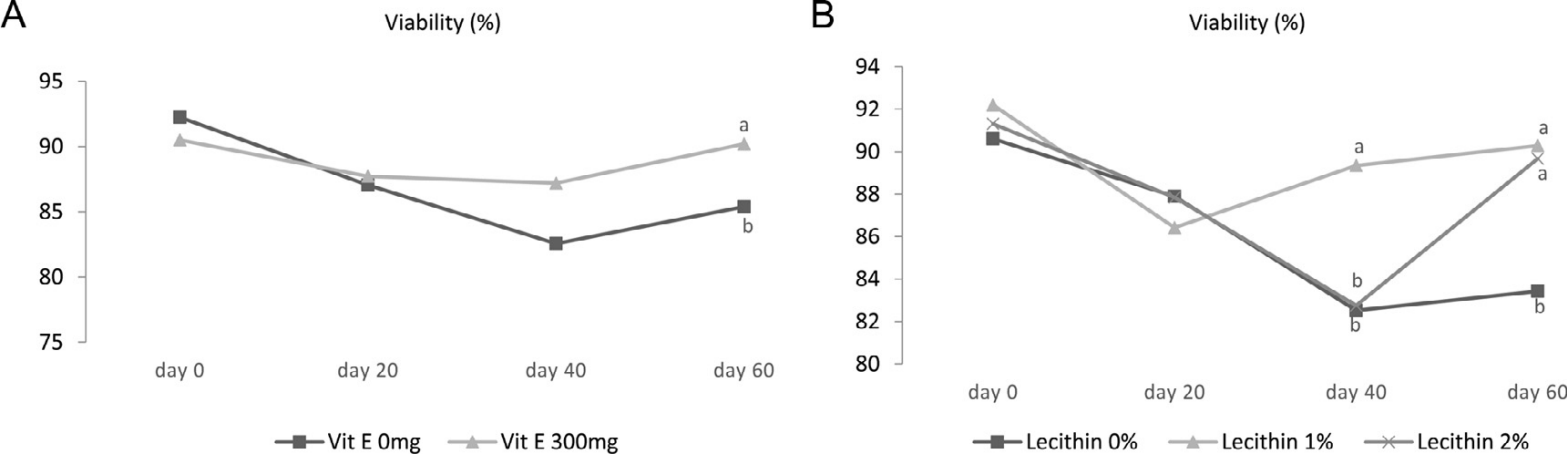
Effect of vitamin E × time interaction (A) and vitamin × time interaction (B) on viability in 45-wk-old Hubbard M99 Line A grandparent roosters fed diets for 60 d. Viability was measured by Annexin V-FITC kit. Superscripts on different means within each day differ significantly at *P* ≤ 0.05.

Effects of vitamin E and lecithin on sperm membrane integrity, sperm viability, sperm DNA fragmentation, abnormal morphology in sperm, and semen malondialdehyde (MDA) are presented in Table 4. The plasma membrane integrity in the sperm that received a diet containing 1% lecithin with vitamin E was significantly higher than all other treatments except the treatment containing 2% lecithin with 300 mg/kg of vitamin E (*P* < 0.05). Moreover, data on the interaction effect of lecithin × time reveals a significant effect of lecithin addition on increasing membrane integrity at 40 and 60 d of the experiment (*P* < 0.01) (Figure 4). A roughly similar trend was observed for viability and 1% lecithin + vitamin E showed a significant improvement (*P* < 0.01). Interaction effect of lecithin × time and vitamin × time indicated that sperm viability on d 60 of the experiment increased due to the addition of vitamin E (Figure 5B) as well as 2 levels of 1 and 2% lecithin (Figure 5B) (*P* < 0.01). All 3 treatments containing vitamin E demonstrated lower levels of MDA than the other treatments (*P* < 0.05). The study of the main effects also showed that different levels of lecithin had no effect on MDA level but supplementing 300 mg of vitamin E per kg of diet reduced the MDA level (*P* < 0.05).

**Table 4:** Effect of lecithin and vitamin E on DNA fragmentation, membrane integrity, morphology, viability, and malondialdehyde (MDA).

		DNA fragmentation (%)	Membrane integrity (%)	Morphology (%)	Viability (%)	MDA (nmol/mL)
Treatments^1^						
Lecithin, %	Vitamin E, ppm					
0	0	6.35	82.86^b^	12.53	86.50^b^	2.52^a^
0	300	6.48	82.33^b^	12.45	86.59^b^	2.22^b^
1	0	6.84	83.49^b^	12.31	87.68^b^	2.59^a^
1	300	5.54	88.13^a^	11.82	92.64^a^	1.98^c^
2	0	6.46	84.10^b^	11.59	87.52^b^	2.55^a^
2	300	6.72	85.93^ab^	11.55	88.92^b^	2.01^bc^
SEM Pooled		1.089	3.056	1.110	1.649	0.149
Main effects						
Lecithin, %						
0		6.41	82.60^b^	12.49	86.55^b^	2.37
1		6.19	85.81^a^	12.07	90.31^a^	2.30
2		6.59	85.02^a^	11.57	88.23^b^	2.28
Vitamin E, ppm						
0		6.55	83.48^b^	12.14	87.24^b^	2.55^a^
300		6.25	85.46^a^	11.94	89.53^a^	2.07^b^
*P* value						
Lecithin		0.758	0.003	0.235	0.0004	0.142
Time		0.597	0.002	0.841	<0.0001	<0.0001
Vitamin E		0.500	0.009	0.642	0.002	<0.0001
Lecithin × Time		0.223	0.003	0.230	0.0003	0.156
Lecithin × Vitamin E		0.296	0.023	0.886	0.009	0.014
Vitamin E × Time		0.148	0.278	0.514	0.0009	<0.0001
Lecithin × Vitamin E × Time		0.462	0.427	0.347	0.308	0.024

Data are presented as means ± SEM (n = 6 roosters/each treatment).

Superscripts with different means within column differ significantly at *P* ≤ 0.05.

1 Roosters received their diets at 45 wk of age for 60 d based on experimental design as follows: 1) basal diet, 2) basal diet supplemented with 300 mg/kg vitamin E, 3) basal diet supplemented with 1% lecithin, 4) basal diet supplemented with 1% lecithin along with 300 mg/kg vitamin E, 5) basal diet supplemented with 2% lecithin, and 6) basal diet supplemented with 2% lecithin along with 300 mg/kg vitamin E. Sperm Parameters: DNA Fragmentation (Acridine Orange test), plasma membrane integrity (hypo osmotic swelling test), morphology (Hancock solution), viability (Annexin V/Propidium iodide), and lipid peroxidation index (Malondialdehyde) were assessed.

Sperm viability and plasma membrane integrity are important factors contributing to sperm fertilization potential because the plasma-membrane damage may cause changes in the DNA and sperm proteins, preventing sperm fertilization (Emamverdi et al., 2013). Our findings in this part are in a logical trend with the other discussed parameters, indicating that there is a positive relationship between motility, viability, and plasma membrane integrity.

Soybean lecithin contains of alpha, gamma, and delta tocopherols (Wang and Wang, 2008). The main antioxidant mechanism of lecithin might be attributed to the synergistic effect between amino-alcohol phospholipids as well as gamma and delta tocopherols (Judde et al., 2003). Improvement in plasma membrane integrity in the current study are in line with the study of Surai et al. (2019) who reported that the inclusion of 200 mg of vitamin E/kg into rooster diets significantly improved sperm membrane integrity. Moreover, Zanussi et al. (2019) assessed the effects of dietary flaxseed oil and vitamin E on sperm characteristics and stated that the supplementation of flaxseed oil and vitamin E into the diet of aged roosters improved membrane functionality and viability.

Sperm DNA fragmentation is closely linked to abnormal sperm morphology and is one of the most widely used approaches to ensure sperm quality (Avendano et al., 2009). In the current study, the addition of vitamin E and soybean lecithin in the diet of roosters yielded no significant effects on DNA fragmentation or abnormal sperm morphology. Our findings are not in accordance with Adabi et al. (2011) reported that adding 150 mg of vitamin E/kg into the diet of quail decreased the percentages abnormal sperm, which may be attributed to an increase in the concentration of alpha-tocopherol in the semen and a threefold decrease in its lipid peroxidation. In the same trend, Sanocka and Kurpisz (2004) showed that adding vitamin E to semen samples decreased the percentage of abnormal sperm and increased fertility. This discrepancy might be related to the difference in diet formulation, species, age of the birds, and concentration of vitamin E.

**Figure 6:**
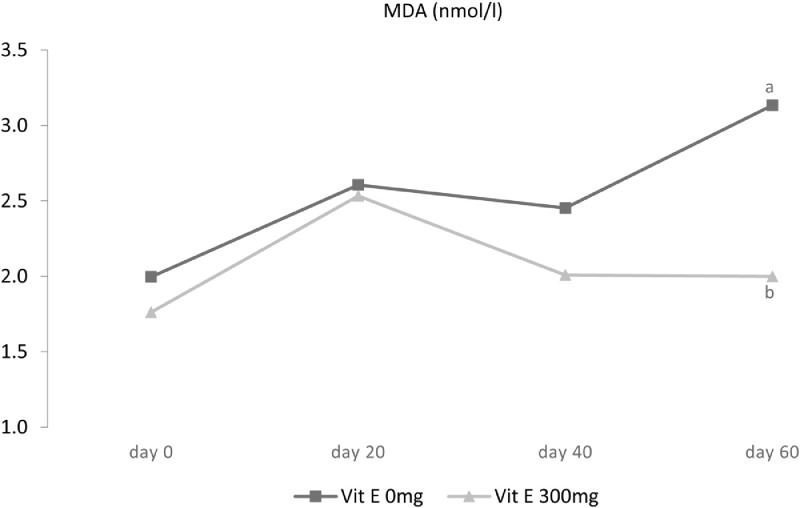
Effect of vitamin E × time interaction on seminal MDA in 45-wk-old Hubbard M99 Line A grandparent roosters fed diets for 60 d. MDA was measured by of thiobarbituric acid. Superscripts on different means within each day differ significantly at *P* ≤ 0.05.

The lowest concentration of MDA was found for roosters fed a combination of lecithin (1 or 2%) and vitamin E (*P* < 0.05). Figure 6 shows the interaction effect of vitamin × time on MDA. It appeared that on the last day of the experiment, vitamin E greatly decreased the concentration of MDA in the semen (*P* < 0.01). MDA is able to penetrate through the plasma membrane and change the symmetry in the lipid composition and consequently interact with DNA that eventually may cause the DNA fragmentation (Ernster, 1993). Based on the findings of this study, the concentration of MDA in the semen of roosters receiving diets supplemented with vitamin E was significantly lower than other treatments (*P* < 0.05); however, lecithin supplementation did not affect the concentration of MDA (*P* > 0.05). The study by Surai et al. (2019) found a negative correlation between sperm lipid peroxidation and vitamin E concentration in semen which is in accordance with our current findings. Higher levels of vitamin E were found to be associated with lower levels of peroxidation in sperm. Furthermore, our findings related to the lipid peroxidation are reflected in study reported by Behnamifar et al. (2020) who reported that 40 mg alpha-lipoic acid (**ALA**) as a feed supplement decreased seminal MDA concentration (*P* < 0.05).

## CONCLUSION

This study found that vitamin E and soybean lecithin improved the quality and motility parameters of rooster sperm, especially sperm viability and membrane integrity. Soybean lecithin at 1% level provided better preservation of sperm quality. Given the positive interaction effects between vitamin E and soybean lecithin, it seems that using a combination of both supplements can improve fertility related parameters in Hubbard “M99” Line A grandparent rooster. It must be noted that duration of feeding supplement is so important, and roosters need to be supported by the supplement at least for one cycle of spermatogenesis. Fertility trial needs to be performed to validate in vitro findings.
